# Surgical management of mediastinal mature cystic teratoma of the elderly remaining asymptomatic

**DOI:** 10.1186/s13019-024-02503-6

**Published:** 2024-01-28

**Authors:** Badreddine Belayachi, Hicham Fenane, Yassine Msougar

**Affiliations:** Department of Thoracic surgery, Mohammed VI University Hospital, Marrakesh, Morocco

**Keywords:** Mediastinal germ cell tumor, Mature teratoma, Mediastinal cyst, Open thoracotomy

## Abstract

**Background:**

Mediastinal teratoma is an uncommon disease, nevertheless they represent the most common mediastinal germ cell tumors. It may grow silently for several years and remain undiagnosed until the occurrence of a complication.

**Aim:**

The main aim of this article is to illustrate the silent evolution of an anterior mediastinal teratoma for over 70 years without presenting any notable complications.

**Case presentation:**

We present the case of a 70-year-old female, treated for hypertension referred to our department for managing a voluminous mediastinal mass, discovered fortuitously by a general practitioner in a chest X-ray. The anamnesis didn’t relate any chest pain, cough, dyspnea nor hemoptysis. The clinical examination, in particular pleuropulmonary, was unremarkable. The workup (Chest X-Ray and CT scan) demonstrated a voluminous pleural mass at the expense of the right mediastinal pleura, rounded in shape, with calcified wall and fluid content. Blood tests did not demonstrate eosinophilia, and hydatid IgG serology was negative. serum human chorionic gonadotropin (hCG) and alpha fetoprotein (AFP) levels were found to be normal. The patient subsequently underwent a right posterolateral thoracotomy with resection of the lesion. The mass was dissected very carefully and then resected in toto. The macroscopic and microscopic histological examination demonstrated a mature cystic teratoma. Surgical resection was an adequate treatment and the prognosis was excellent for the patient.

**Conclusion:**

Cystic mature teratomas are rare thoracic tumors, often recognized by radiological examination. This article relates the silent evolution that a teratoma could have, and the late appearance of symptoms that it could have.

## Introduction

Teratomas are the most common mediastinal germ cell tumors (60-70%). They’re known to be the most common extragonadal germ cell tumors in prepubertal and post pubertal patients, regardless of sex, representing 15% of all mediastinal masses in adults and 25% in children [1].

These tumors are characterized by the formation of variable somatic tissues with random distribution, originating from two or three germ layers [2]. Three histological types were found: mature, immature, or with malignant transformation. Mature teratomas constitute 60% of germinal mediastinal tumors. They usually have a cystic appearance and are usually not accompanied by symptoms. Immature teratomas are distinguished from mature teratomas by the additional presence of fetal-like tissue without specific tissue organization.

Histogenesis refers to the migration theory (migration of primordial germ cells from the extraembryonic mesoderm to the genital ridges) as well as the thymic “totipotent cell” theory. However, no cytogenetic abnormalities in teratomatous germ cell tumors have been proven.

This article marks the difficulties that could be encountered with having a preoperative histological diagnosis for mediastinal cystic masses, particularly when asymptomatic, and the important of postoperative diagnosis in such cases.

## Patient and observation

We present the case of a 70-year-old female, with a history of arterial hypertension treated with ACE inhibitors and thiazides for 19 years, with no other personal of family history, who consulted in our department for a stage I shortness of breath (Sadoul staging) evolving for a year, without chest pain, cough, or hemoptysis, the anamnesis also relates to the notion of hydatid contagion consisting of a close contact with a dog.

**Fig. 1:**
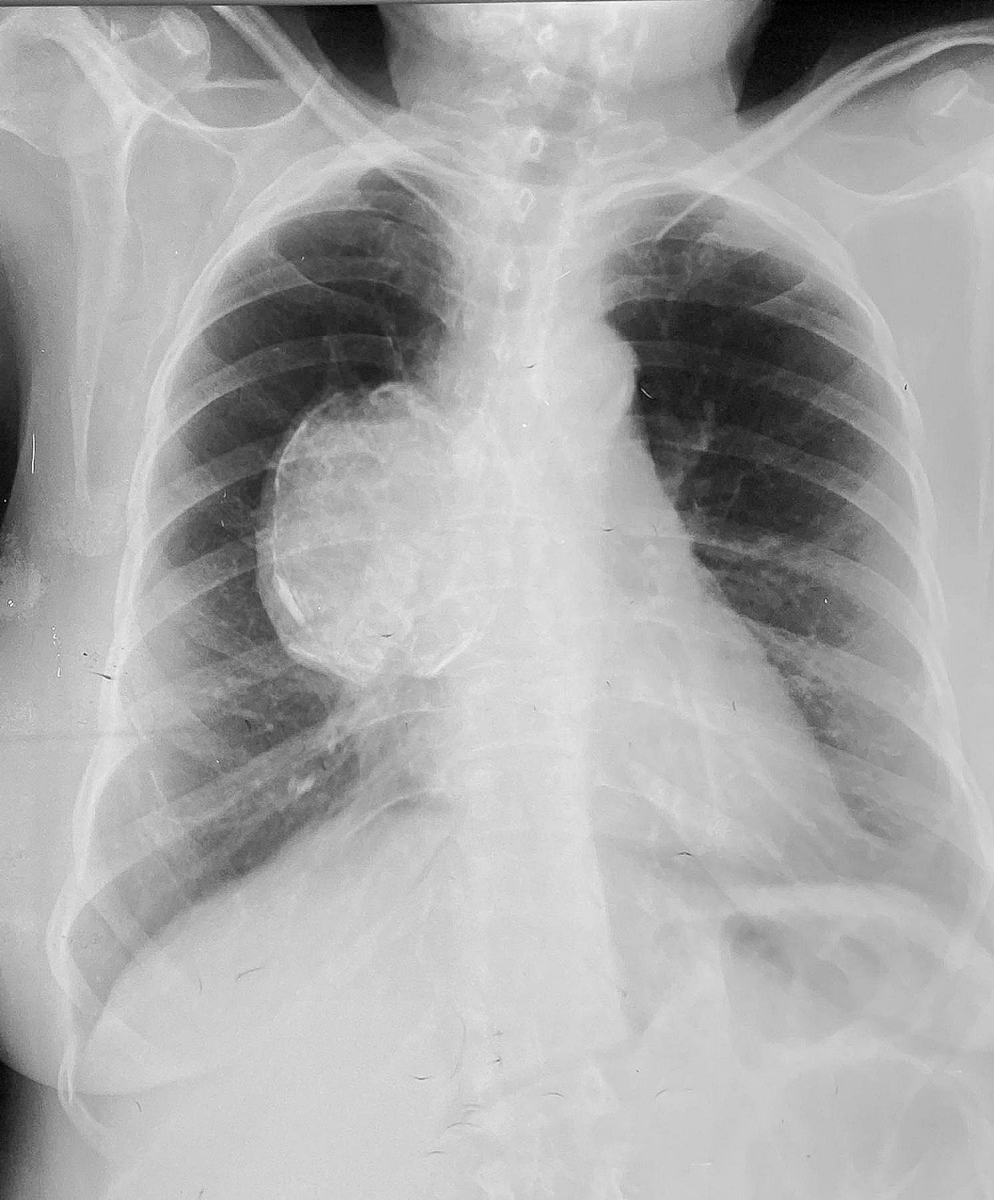
Preoperative chest Xray showing a round well limited opacity, with calcified limits

**Fig. 2:**
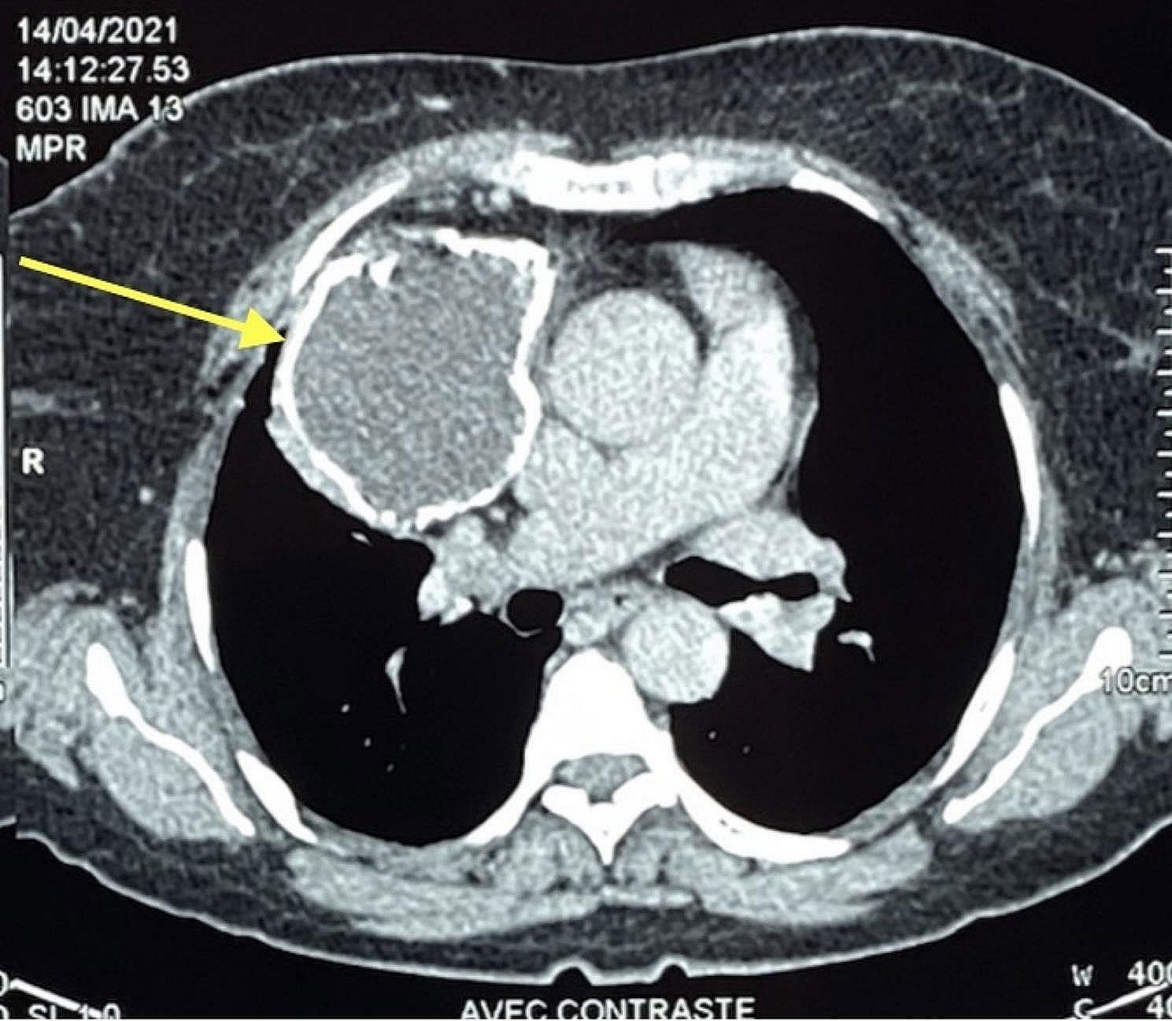
CT scan image of the right mediastinal mass (Yellow arrow)

The general physical evaluation was within normal limits and the respiratory system examination was unremarkable. Chest X-Ray revealed a round homogenous opacity in the right hemithorax, with calcified wall and hilar position in contact with right mainstem bronchus and heart but with no mass effect or shift of the mediastinum. (Fig. 1) and the chest CT scan (Fig. 2), showed a Pleural mass involving the right mediastinal pleura, rounded in shape, with calcified walls and hypodense liquid content, not enhanced after contrast injection, measuring 78 × 64 × 83.4 mm. Internally: it comes into contact with the left atrium, the ascending thoracic aorta, the superior vena cava (SVC), and the dilated right internal mammary vein. Externally: it comes into contact with the anterior arches of the 2nd and 3rd right ribs without detectable bone lysis. It also displaces the middle lobe with linear atelectasis in the corresponding area. No additional imaging was performed, for instance brain CT was not performed since patient showed no neurological signs.

Blood tests did not demonstrate eosinophilia, and hydatid IgG serology was negative. Renal and hepatic function was normal as well as a complete ionogram. Tumor markers such as serum human chorionic gonadotropin (hCG) and alpha fetoprotein (AFP) levels were measured and were found to be normal. Autoimmune disease such as myasthenia was evaluated, and Anti-acetylcholine receptor antibodies were negative.

Bronchoscopy with liquid aspiration was performed in the search of scolex, but came back negative as well. Fine needle aspiration cytology was discussed with radiology team but was judged too risky due to the intimate contact of the mass with vital mediastinal structures.

**Fig. 3:**
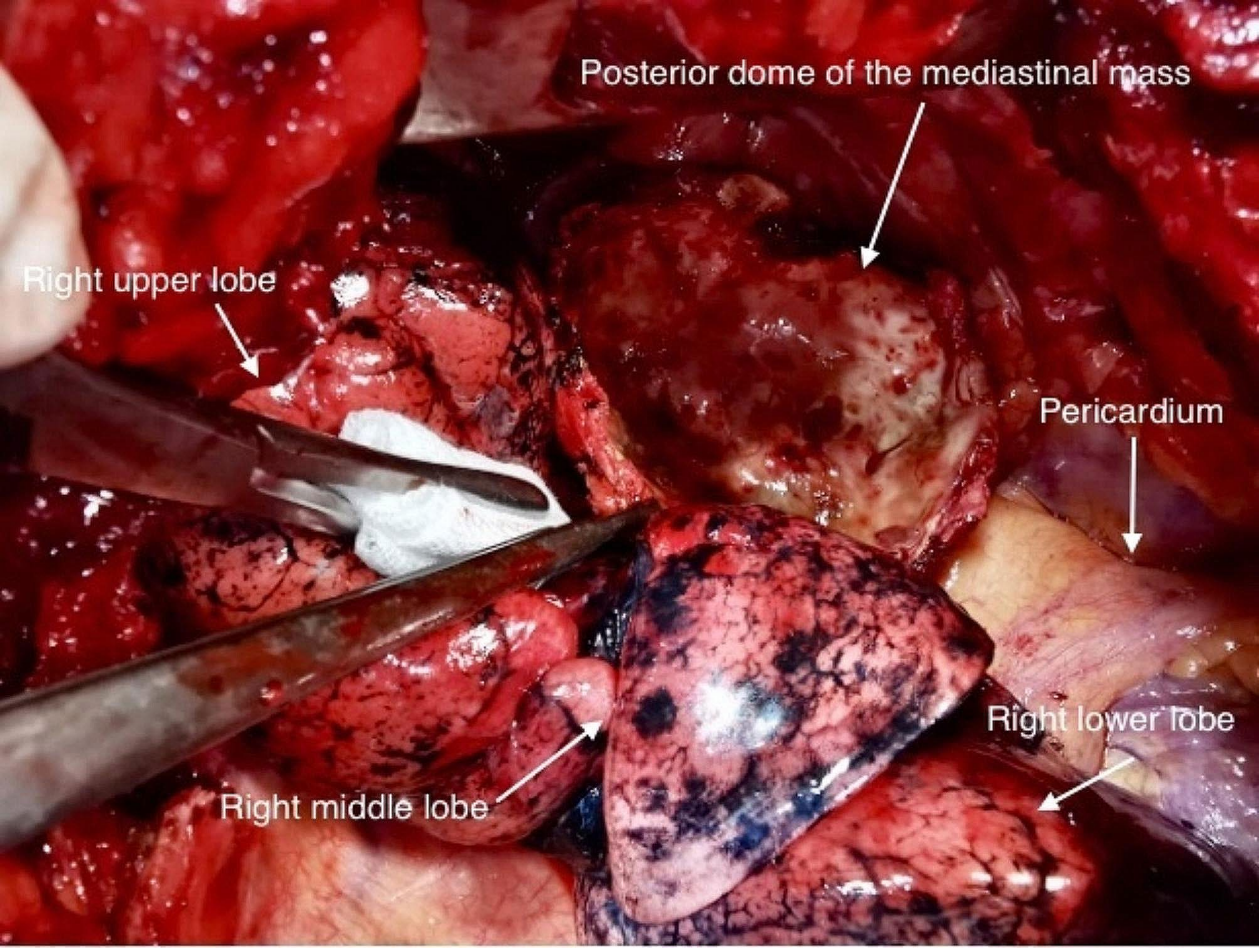
Open chest view of the mass (right posterolateral thoracotomy)

Therapeutic decision was surgical removal of the tumor. The patient subsequently underwent right posterolateral thoracotomy with resection of the lesion. VATS surgery could not be performed due to financial difficulties, since the patient couldn’t afford the cost. Anesthesia was general and ventilation through a Robertshaw double-lumen endotracheal tube. The mass was strongly adherent to the SVC, and was dissected very carefully, the posterior wall was too adherent to be dissected and removed, anterior wall as well as cyst content were removed (Fig. 3). The macroscopic histological examination demonstrated a cystic formation, divided into 2 fragments, measuring 10 × 9 × 6 cm and weighing 100 g, was received. The wall was thick, firm, and irregular. On sectioning, it appeared beige in color with areas of fibrous and hemorrhagic changes, as well as the presence of hair. Microscopy identified a cystic wall lined by a squamous epithelial lining, accompanied by mild orthokeratotic keratosis. The stroma was fibrous and contained a few hair follicles and rare sebaceous glands. Additionally, regular adipose tissue was present. There was no evidence of tumorous proliferation. In conclusion, the morphological aspect was that of a mature cystic teratoma.

**Fig. 4:**
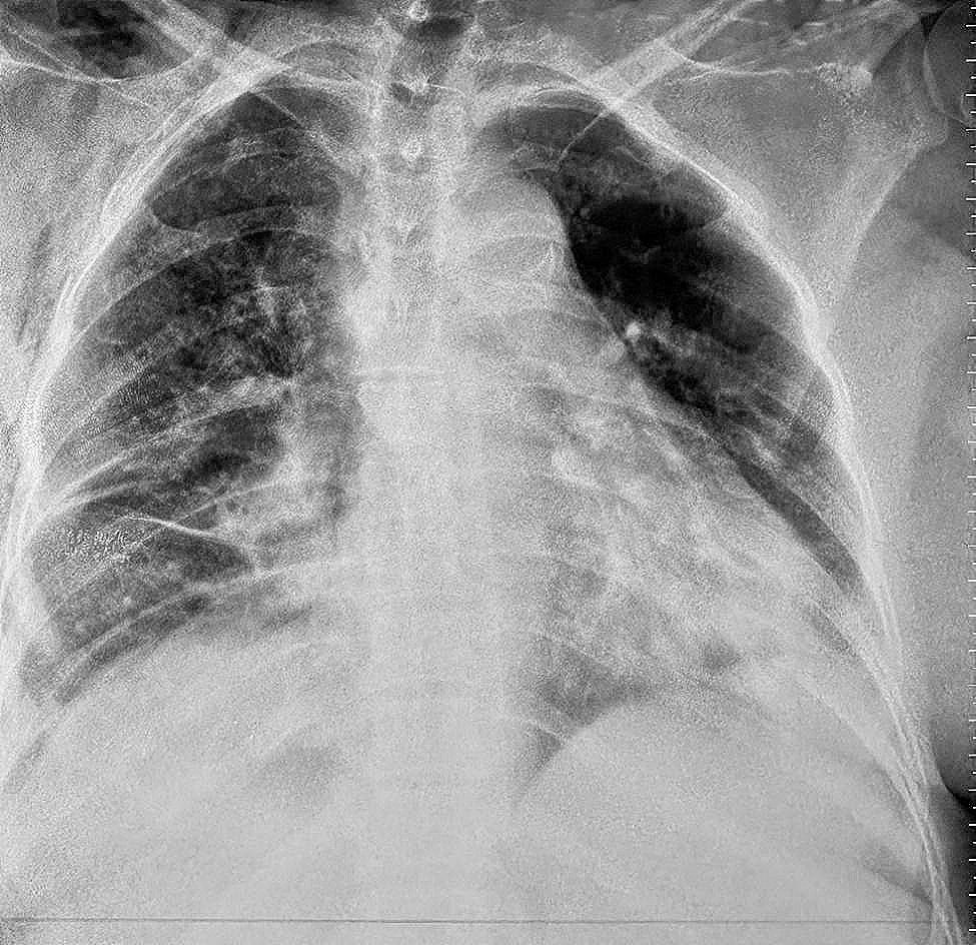
Postoperative Chest Xray showing the disappearance of the opacity

Postoperative evolution did not show any complications (Fig. 4), in particular no bleeding was noted, pain was managed with paracetamol, nefopam chlorhydrtate and NSAIDs. Chest tube was removed and the patient was discharged five days after surgery, and the follow-up examinations showed no abnormalities and x-rays at 1, 3 and 6 months and the disappearance of the opacity and its calcified wall.

## Discussion

Mediastinal teratomas are the most common mediastinal germ cell tumor, with no significant sex difference, unlike other mediastinal tumors. The mediastinum is the most common extragonadal site of teratomas [3]. Mostly diagnosed in the adolescent/young age group, only 2% of diagnosed patients are 55–60 years old [2]. Our patient was 70 years old.

Several theories suggest the origin of the teratoma, including the theory of the migration of primordial germ cells from the extraembryonic mesoderm to the genital ridges or even the thymic “totipotent cell” theory, in which mediastinal teratoma is derived from the spontaneous vascular development of some potential stem cells shed during the development of thymus primordia at the embryonic stage [4]. During the fourth and fifth weeks of gestation, these cells develop among the endodermal cells in the yolk sac and migrate to the gonadal ridges. However, some cells may miss their designated destination and cause teratomas that arise anywhere between the brain and the coccygeal area, typically in the midline [5].

Teratomas have diverse histological appearances, with tissues randomly distributed based on their level of differentiation. They are categorized into two main types: mature and immature [6]. Mature teratomas, accounting for 60-70% of germinal mediastinal tumors, consist of well-differentiated benign tissues, often appearing cystic and asymptomatic for extended periods. Immature teratomas contain fetal-like tissue without specific organization or neuro-endocrine elements [7].

Teratomas are often hard to detect when small and asymptomatic [4]. Symptoms typically arise due to the mass’s pressure on surrounding structures, causing issues like dyspnea and chest tightness [8]. In some cases, external puncture can release enzymes, leading to complications like pleural effusion or pericardial effusion [2]. In this particular case, the patient experienced stage I dyspnea without additional symptoms.

Imaging is a strong element in the diagnosis of a teratoma. Standard chest X-ray remains essential, particularly for asymptomatic patients. It usually shows a large, rounded or ovoid tumor, with well-defined limits, often polylobed, in most of the cases located in the anterior mediastinum, and often lateralized to one hemithorax. CT remains the most efficient radiological exam, the classic finding is that if a well-circumscribed unilocular or multilocular anterior mediastinal mass, cystic or cystic-solid with a heterogeneous density inside the cyst representing fluid, soft tissue and fat attenuation [6]. The CT of our patient showed a round mass at the expense of the right mediastinal pleura, with calcified wall and fluid content (Fig. 2). Teratomas on MRI display varying internal elements, resulting in heterogeneous signal intensity. A chemical shift MRI is frequently used to distinguish teratomas from thymomas and lymphomas, as well as the presence of fat-fluid levels, which are nearly diagnostic for teratomas. An increase in the signal intensity is typically seen in lymphomas and thymomas [6]. Our patient could not undergo an MRI due to financial difficulties.

Biological exploration of mediastinal teratoma requires monitoring of serum tumor markers, a high serum AFP or β-HCG level could indicate a malignant component of the teratoma, such as an embryonic carcinoma, or a choriocarcinoma [9]. Here, hCG and AFP were found to be at normal levels. Although the radiological features were not in favor of a hydatid origin, this one could not be totally ruled out since our country represents an endemic region, and since calcified hydatid cysts in the mediastinum have been reported in our country [10].

Mediastinal mature teratomas can be managed with complete surgical resection, open thoracotomy is still the first choice regarding voluminous or malignant tumors, VATS is widely considered a feasible choice, but tumor size and eventual complications, such as intraoperative bleeding and strong adhesions, have been behind an important conversion rate to open thoracotomy [9]. Our patient underwent an open thoracotomy resection, decision was taken since surgery is treatment of choice and since we had no other mean to have histology sample, the presence of malignant cells would indicate chemotherapy. Difficulties were indeed encountered, such as a 200 ml bleeding and strong adhesions, in particular to the SVC. (Fig. 3).

Benign cystic teratomas typically exhibit a favorable prognosis following surgical treatment, although there is a slight risk of recurrence within a 2 to 10-year timeframe. In contrast, cystic teratomas with malignant transformation display varying outcomes, influenced by factors such as the stage, growth pattern, and the extent of vascular invasion within the tumor [11].

## Conclusion

In conclusion, this case report highlights the remarkable 70-year asymptomatic evolution of an anterior mediastinal teratoma. It underscores the importance of early detection through radiological examination and shares valuable insights on managing surgical challenges. This unique case contributes to our understanding of mediastinal teratomas and offers practical lessons for healthcare professionals.

## Data Availability

The data used to support the findings of this study are available for review.
